# Supplementary material for: Modelling Skylarks (Alauda arvensis) to Predict Impacts of Changes in Land Management and Policy: Development and Testing of an Agent-Based Model
Source: PLoS One. 2013 Jun 6;8(6):e65803. doi: 10.1371/journal.pone.0065803 (PMC3675089; doi:10.1371/journal.pone.0065803)
Supplement: Supporting Information S4 — The skylark ODdox as a zipped archive. (ZIP) [file pone.0065803.s004.zip › Skylark_ODdox/globals_type.html]

ALMaSS Skylark ODdox: File Members


ALMaSS Skylark ODdox
 2.0


- Main Page
- Related Pages
- Classes
- Files

- File List
- File Members

- All
- Functions
- Variables
- Typedefs
- Enumerations
- Enumerator
- Macros

- AnsiString
  : populationmanager.h
- base\_generator\_type
  : BoostRandomGenerators.h
- distribution\_type
  : BoostRandomGenerators.h
- gen\_type\_int
  : BoostRandomGenerators.h
- LE\_Signal
  : elements.h
- PointerInt
  : ALMaSS\_Setup.h
- Skylark\_ClutchList
  : skylarks\_all.h
- Skylark\_FemaleList
  : skylarks\_all.h
- Skylark\_MaleList
  : skylarks\_all.h
- Skylark\_NestlingList
  : skylarks\_all.h
- Skylark\_PreFledgelingList
  : skylarks\_all.h
- TListOfAnimals
  : populationmanager.h
- uint32
  : ALMaSS\_Setup.h
- uint64
  : ALMaSS\_Setup.h


- Generated on Thu Jan 10 2013 13:15:39 for ALMaSS Skylark ODdox by
   1.8.1.1
